# The Emergence of the Family Scirtidae (Insecta: Coleoptera) in Lotic Karst Habitats: A Case Study over 15 Years

**DOI:** 10.3390/insects15040226

**Published:** 2024-03-26

**Authors:** Ana Klarin, Marija Ivković, Vlatka Mičetić Stanković

**Affiliations:** 1 Lojenov prilaz 4, 10000 Zagreb, Croatia; anaklarin99@gmail.com; 2 Division of Zoology, Department of Biology, Faculty of Science, University of Zagreb, Rooseveltov trg 6, 10000 Zagreb, Croatia; marija.ivkovic@biol.pmf.hr; 3 Croatian Natural History Museum, Demetrova 1, 10000 Zagreb, Croatia

**Keywords:** biology, ecology, freshwater, long-term study, phenology, diversity, water beetles

## Abstract

**Simple Summary:**

This study is a case study on the diversity patterns, population aspects, emergence and ecological drivers of the so-far poorly studied water beetle family Scirtidae. Although they can be very abundant in karstic lotic freshwater habitats, there are still insufficient data on their biology and ecology. The family was studied over a period of 15 years in specific lotic habitats of springs and tufa barriers in the karst freshwater ecosystem of Plitvice Lakes NP, Croatia. Scirtidae showed a longitudinal gradient, as the genus *Elodes* was recorded only in the spring area, while the genus *Hydrocyphon* was found only in downstream sites with tufa barriers. Their emergence showed a seasonal character, with males dominating earlier in the season and females appearing later. The type of substrate determined their distribution, with a preference for bryophytes. Their abundance and emergence were primarily determined according to the parameters that showed the greatest fluctuations over the 15-year period: water discharge, water temperature and oxygen saturation. Compared to previous results from similar studies on other insect groups, our results clearly show the high potential of the family Scirtidae as indicators of habitat quality.

**Abstract:**

Due to ongoing changes and a decline in biodiversity, science today should rely on long-term species-based ecological studies. We have conducted a long-term ecological dynamics study on the water beetle family Scirtidae, which, although it is very abundant in benthic communities, is still poorly studied. The main objective of this study was to investigate the population aspects (composition, diversity, sex ratio) and ecological aspects (emergence patterns, seasonal dynamics and preferences for environmental factors) of the family Scirtidae over 15 years in Plitvice Lakes NP, Croatia. The study was conducted at three sites and in five different substrate types. A total of three taxa with different distributions were recorded in the study area: *Hydrocyphon novaki* and *H. deflexicollis* on the tufa barriers and the *Elodes* sp. in the spring area. The sex ratio was in favour of males in spring and early summer, while it shifted in favour of females towards the end of autumn. The abundance and emergence of the family were primarily determined by the environmental parameters which showed the greatest fluctuations over a period of 15 years: water temperature, water discharge and oxygen saturation. Our results clearly show that Scirtidae can be used as indicators of stream zonation and habitat quality. Based on the methodology and the results of this study, we conclude that Scirtidae should be used in future monitoring and protection measures in karst freshwater habitats in southeastern Europe.

## 1. Introduction

Water beetles are an ecological group of Coleoptera that is connected with water in at least one stage of their development [1]. They have colonised numerous types of aquatic habitats, with the exception of marine ones [2]. More than 13,000 species of water beetles have been described worldwide so far [2,3]. The families with the highest species richness are Dytiscidae and Hydrophilidae, followed by Elmidae, Hydraenidae, Gyrinidae and Scirtidae. They are used as indicators of water quality and the ecological functionality of water ecosystems [4], regional biodiversity [5,6] and ongoing climate change [7].

The family Scirtidae is one of the most species-rich families of water beetles, with more than 1800 species described worldwide to date [8]. Jäch [1] defines them as false water beetles, as the adult stage emerges on land. Scirtids are distributed in all zoogeographical regions, with the greatest diversity found in tropical and temperate regions in the Northern Hemisphere [9]. Although they can dominate benthic invertebrate communities [10,11], data on their biology and ecology are very scarce. Recently, the family came into focus when Nadein et al. [12] presented their detailed study on the distinct jumping mechanism of the marsh beetle *Scirtes hemisphaericus* (Linnaeus). The distinctiveness of the family is also highlighted by the copulation of the genera *Contacyphon* and *Hydrocyphon*, in which females penetrate males with a unique structure (*prehensor*) to extract spermatophores, which is very rare in the animal world [13,14,15].

Southeastern Europe is considered a hotspot of biodiversity [16,17], especially the Dinaric karst region. This region extends from northeastern Italy to Albania [18,19], where many endemic species have been recorded so far [20,21,22]. Karst freshwater habitats are particularly affected nowadays, as climate change, water use and spring water diversion seriously alter their morphology and hydrology (e.g., [19,23]). Plitvice Lakes NP is one such sensitive ecosystem, known for numerous springs, a barrage lake system and tufa barriers. Tufa formation depends strongly on the stability of self-biodynamic processes, which are extremely sensitive to anthropogenic pressure [24].

In Croatia, 7 genera and 15 species of scirtids have been discovered so far [11,25,26,27,28,29,30]. Mičetić Stanković et al. [11,31] studied them as part of the water beetle community in karst lotic habitats and provided the first data on the biology and ecology of the family in these habitats. They have found that scirtids are very abundant in tufa barriers and indicate the zonation of the stream, with its distribution defined by the current velocity and canopy cover. Many studies have focused on the emergence of aquatic insects in karst freshwater habitats in southeastern Europe so far: Diptera (e.g., [32,33]), Trichoptera (e.g., [34]), Ephemeroptera (e.g., [35]) and Plecoptera (e.g., [36]). The latter studies have found that water temperature and photoperiod are key factors in the emergence patterns of aquatic insects. On the contrary, the emergence of the family Scirtidae has never been studied before. Studies that focus on long-term data are scarce, with only a handful undertaken on aquatic insects [37], with just a few in karstic habitats [35,38,39,40]. Long-term studies are the only type of studies that can tackle the influence of climate change on these communities, especially when it comes to pristine habitats, such as Plitvice Lakes NP [37,38].

The present study in a lotic karst freshwater system aimed to fill the knowledge gap on the population and emergence patterns and ecological drivers of the family Scirtidae. The objectives of the present study were to analyse its (i) diversity and composition; (ii) sex ratio; (iii) and emergence patterns and (iv) the impact of environmental characteristics on Scirtidae. This study will contribute not only to our overall knowledge on freshwater biodiversity but also to the conservation of exceptional karst habitats.

## 2. Material and Methods

### 2.1. Study Area

**Figure 1:**
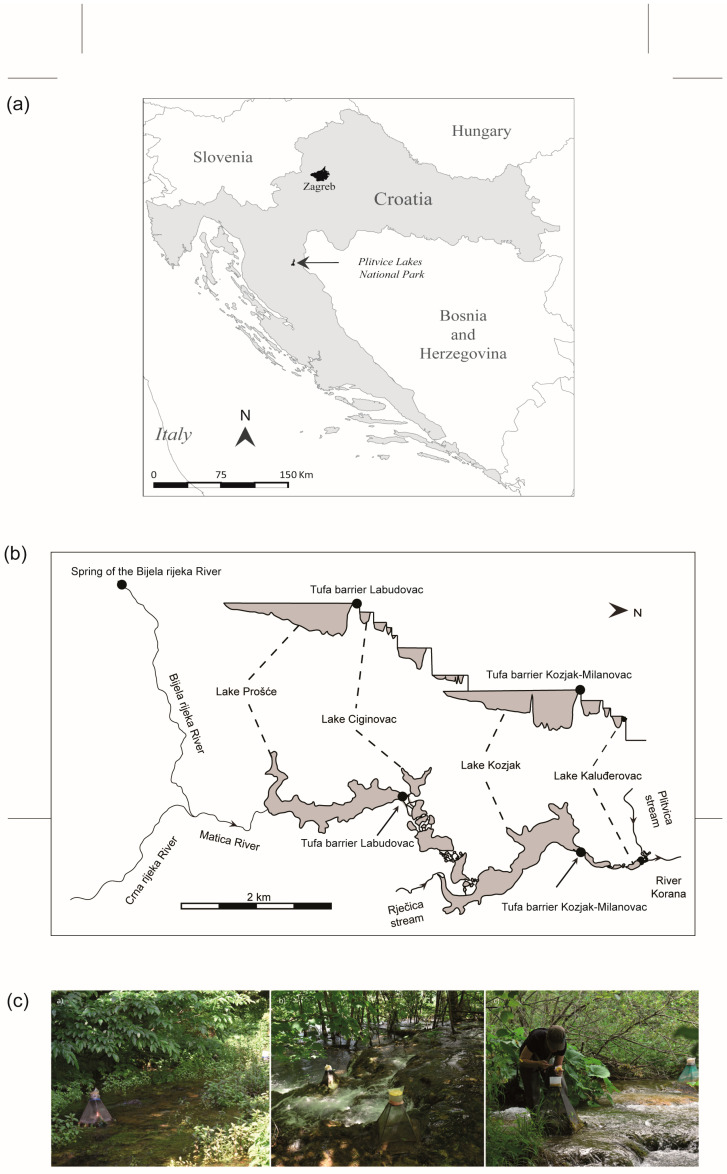
Map of the study area: (**a**) position of Plitvice Lakes NP (indicated with arrow); (**b**) three sampling sites (shown with black dots) in Plitvice Lakes NP; (**c**) some of the emergence traps (from left to right): spring of the Bijela river—tufa barrier Labudovac—tufa barrier Kozjak–Milanovac. Tufa barriers are also shown in the lateral profile (**b**), as they are located between adjacent lakes.

Plitvice Lakes National Park is world-famous for its cascade system of 16 lakes connected by beautiful waterfalls and tufa barriers. It was designated as a national park back in 1949 and internationally recognised as a UNESCO World Heritage Site in 1979 [24]. An area of 295 km^2^ is located in the Dinaric continental sub-ecoregion with a moderately warm, rainy and snowy climate [24] (Figure 1). The greatest precipitation falls in the autumn and winter months. The air temperature varies greatly and can drop to −25 °C in winter, while it can exceed 30 °C in summer [41,42,43]. The lakes are mainly fed by the Matica River, which was formed by the confluence of two mountain rivers, the Bijela rijeka river and the Crna rijeka river [24]. The geological bedrock consists of carbonates and dolomites, which form a cavernous, shallow karst rich in springs and streams [44]. The unique hydrogeological characteristics of the park result in a cascade system where the lower lakes are buried in limestone, while the upper lakes are elevated by tufa barriers. The water in the catchment area is rich in calcium salts and is limited in organic matter with a pH of around 8. These conditions, combined with the presence of algae and bryophytes, favour the formation of tufa [44,45]. Tufa barriers are geological formations characterised by a porous and layered structure. These barriers can vary in size and shape, ranging from small dams to extensive formations, and they play crucial roles in shaping aquatic ecosystems by influencing the water flow and sediment deposition and providing habitats for various plant and animal species [24].

To investigate the emergence patterns of the family Scirtidae, both the spring area and the tufa barriers of the lake system were investigated in the present study: the Spring of the Bijela rijeka river (SBR), the Labudovac tufa barrier (TBL) and the Kozjak–Milanovac tufa barrier (TKM). The characteristics of the sites and the defined substrate types are listed in Table 1.

### 2.2. Sampling and Identification

The emergence patterns and biological and ecological characteristics of Scirtidae were studied over 15 years—from February 2007 to December 2022. The samples from 2015 were unfortunately lost in a terrible earthquake that struck Zagreb in 2020. Emergence traps in the form of four-sided pyramid structures were used, which were set up at three study sites. At the spring of the Bijela rijeka river and the Kozjak–Milanovac tufa barrier, six traps were set throughout the study period. At the Labudovac tufa barrier, six traps were initially set between 2007 and 2011, and in 2012, an additional trap was set, making a total of seven traps. The traps were placed in different microhabitats, depending on the type of substrate and the current velocity (Table 1). Each emergence trap had a height of 50 cm and a base area of 45 cm × 45 cm. The side frames of the traps were covered with a 1 mm thick mesh net, which was securely fixed in the streambed but still allowed the benthos to move freely. Collecting containers were placed at the apex of each trap, filled with a preservative solution of 2% formaldehyde with detergent. Sampling was undertaken monthly, and the collected material was preserved and treated with 80% ethanol. The specimens were identified to the lowest possible taxonomic level following Klausnitzer [45]. Within the family Scirtidae, the genera can be easily distinguished by their external morphological characters, including their antennae, legs, elytra and pronotum. However, identification of the species requires dissection and examination of the genital structures. While most representatives of Scirtidae as well as other beetles can be identified according to the morphology of the male genitalia, the females of the genus *Hydrocyphon* can be successfully distinguished at the species level according to the structure of their reproductive system—the prehensor. This structure is missing in the females of the genus *Elodes*. The voucher specimens are housed in the Croatian Natural History Museum (HPM) in Zagreb.

### 2.3. Abiotic and Biotic Environmental Parameters

The following physical and chemical properties of the water were measured at each sampling site: water temperature and oxygen saturation (using a WTW Oxi 330/SET oximeter, Wissenschaftlich-Technische Werkstätten GmbH, Weilheim, Germany)), pH (using a WTW pH 340i, Wissenschaftlich-Technische Werkstätten GmbH, Weilheim, Germany), conductivity (using a WTW Cond 340i, Wissenschaftlich-Technische Werkstätten GmbH, Weilheim, Germany) and alkalinity (through titration with 0.1 M HCl). Daily average discharge values were provided by the Croatian Meteorological and Hydrological Service. The data on ammonia, orthophosphate and nitrogen were provided by the Plitvice Lakes National Park Public Institution. The current velocity and water temperature were measured at each microhabitat, where emergence traps were placed using a current velocity meter (a P-670-M series, Dostmann electronic, Wertheim, Germany)and a HOBO Pendant Temperature Data Logger (#Part UA-001-XX, Bourne, MA, USA), respectively. Depth was measured using a hand meter on a metal stick.

### 2.4. Data Analysis 

Various biological and ecological aspects of the family Scirtidae in the Plitvice Lakes NP were analysed. This included diversity patterns (number of species, abundance and longitudinal distribution), population aspects (the sex ratio), emergence patterns (which refer to the timing and frequency of emergence and its seasonal dynamics) and the impact of environmental characteristics on Scirtidae (including preferences for current velocity, water depth, temperature, pH, alkalinity, oxygen levels, nutrient availability, conductivity and substrate type). The sex ratio (♂:♀) was defined according to the scirtids’ abundance on a monthly and annual basis [46]. Sex ratio differences were tested using Friedman’s Two-way Analysis of Variance by Rank with Bonferroni correction. The variation in the sex ratio due to substrate type and season was tested using a General Linear Model (GLM). Month was used as a random factor due to temporal pseudo-replication [47]. Prior to the analysis, the biological data were log-transformed.

All the data were tested for normality using D’Agostino’s K-squared test and the Kolmogorov–Smirnov (K-S) test. ANOSIM analysis [48,49] was used to test for differences in the scirtids’ abundance between sampling sites. The influence of the physical and chemical water properties on the scirtids’ emergence was assessed using Spearman’s rank correlation coefficient (ρ) at both the microhabitat and site levels. All the analyses were conducted separately for males and females. The site-level analysis included three taxa and nine physical and chemical parameters (water temperature, pH, alkalinity, oxygen, nutrients, conductivity) for three sites. The microhabitat-level analysis included three taxa and two physical parameters (current velocity and water depth) for a total of 19 microhabitats. Principal component analysis (PCA) was performed on the complete faunal and environmental data for all three sites. The biological data were log-transformed before analysis, while the physical and chemical data were normalised; a full draftsman’s plot was created for interrelation analysis. Ten physical and chemical parameters were used (water temperature, pH, conductivity, oxygen saturation, alkalinity, nitrates, nitrites, orthophosphates, ammonium, water discharge) at three study sites from 2007 to 2022. The analyses were performed using SPSS 17.0 [50] and the software package Canoco for Windows ver. 5.0 [51,52]. Graphs and illustrations were created using Adobe^®^ Illustrator^®^ CS6 [53] and Grapher ™20 [54].

## 3. Results

### 3.1. Faunal Assemblage and Distribution

**Figure 2:**
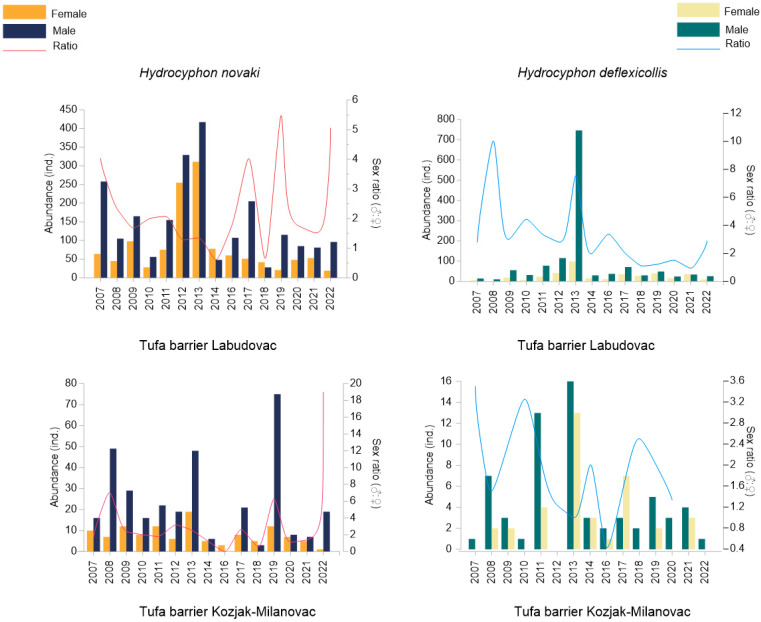
Species abundance and sex ratio of two *Hydrocyphon* species at the tufa barrier Labudovac (**up**) and the tufa barrier Kozjak–Milanovac (**down**) from 2007 to 2022.

During the study period of 15 years, a total of 5845 specimens of scirtids were collected and included in the analyses, representing two genera, *Hydrocyphon* and *Elodes*, and three species: *Hydrocyphon deflexicollis* (Müller), *Hydrocyphon novaki* Nyholm and the *Elodes* sp. When comparing the species abundance between the years of the study, 2013 was singled out as the year with the highest total abundance, with 1669 specimens. The lowest abundance was recorded in 2008, with a total of only 137 specimens altogether. The distribution of the scirtids showed a zonal character, as the genus *Elodes* was only recorded in the spring area, while *Hydrocyphon* was only found at the tufa barriers. The highest abundance was recorded at the Labudovac tufa barrier, with a total of 5230 specimens. At the other tufa barrier, Kozjak–Milanovac, a total of 566 specimens were recorded, while at the spring of the Bijela rijeka river, only 48 specimens were collected (Figure 2). *H. novaki* was more abundant than *H. deflexicollis* throughout the study period, except in 2013, when it was the other way around. *H. deflexicollis* then contributed 845 specimens to the highest total abundance reported for the Labudovac tufa barrier. At the Kozjak–Milanovac tufa barrier, scirtids reached the highest abundance in 2019 with 103 specimens, of which 96 were *H. novaki*. No *H. deflexicollis* specimens were recorded at the same site in 2012. At the spring of the Bijela rijeka river, the abundance of the genus *Elodes* was generally low, as the highest abundance was 20 specimens (in 2020). Moreover, the genus was not recorded at all for a period of eight years (from 2008 to 2016). Thus, due to its very low abundance, *Elodes* was omitted from Figure 2.

**Figure 3:**
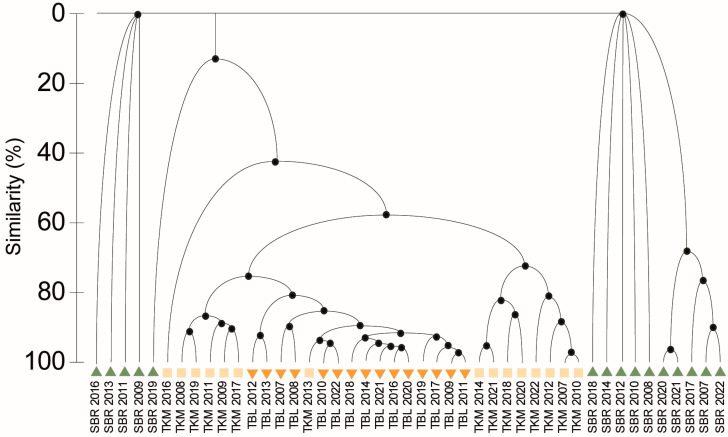
Cluster analysis of microhabitats from 2007 to 2022. Abbreviations: SBR: spring of the Bijela rijeka river; TBL: tufa barrier Labudovac; TKM: tufa barrier Kozjak–Milanovac.

The ANOSIM analysis of the studied sites revealed significant differences in the scirtid abundance (R = 0.635, *p* = 0.01, *p* < 0.05). Cluster analysis of the studied sites at the temporal and spatial scales showed the highest similarity between the years 2009 and 2012 at the Labudovac tufa barrier and the years 2007 and 2010 at the Kozjak–Milanovac tufa barrier. The highest similarity between the tufa barriers was found for the Kozjak–Milanovac tufa barrier in 2013 and the Labudovac tufa barrier in 2010 (Figure 3).

### 3.2. Sex Ratio and Emergence Patterns

Only the genus *Hydrocyphon* was included in the sex ratio analysis, as, surprisingly, only females of the genus *Elodes* were recorded during the whole study period (Figure 2). The sex ratios of the two *Hydrocyphon* species differed at the spatial and temporal scales. The abundance of females and males differed between sites (*Hydrocyphon novaki*, female: *p* = 0.001, *p* < 0.05; male: *p* = 0.001, *p* < 0.05; *H. deflexicollis*, female: *p* = 0.028, *p* < 0.05; male *p* = 0.001, *p* < 0.05). Male specimens of *H. novaki* were more numerous than females, except in 2014 and 2018 at the Labudovac tufa barrier and in 2016 and 2018 at the Kozjak–Milanovac tufa barrier. It is noteworthy that only females were recorded in 2016. The sex ratio was also in favour of males for *H. deflexicollis* at both tufa barriers, except in 2021 (Labudovac tufa barrier) and 2017 (Kozjak–Milanovac tufa barrier), when females were more numerous. Moreover, the sex ratio differed according to the season, so the highest sex ratio value was reported in June, when males were considerably more abundant than females. On the other hand, females were more abundant in the summer months of July and August (*Hydrocyphon novaki*, female: *p* = 0.001, *p* < 0.05; male: *p* = 0.001, *p* < 0.05; *H. deflexicollis*, female: *p* = 0.001, *p* < 0.05; male *p* = 0.001, *p* < 0.05). Water discharge also proved to be an important factor in determining the differences between the abundance of females and males at the study sites (*Hydrocyphon novaki*, female: *p* = 0.001, *p* < 0.05; male: *p* = 0.001, *p* < 0.05; *H. deflexicollis*, female: *p* = 0.029, *p* < 0.05; male *p* = 0.002, *p* < 0.05).

**Figure 4:**
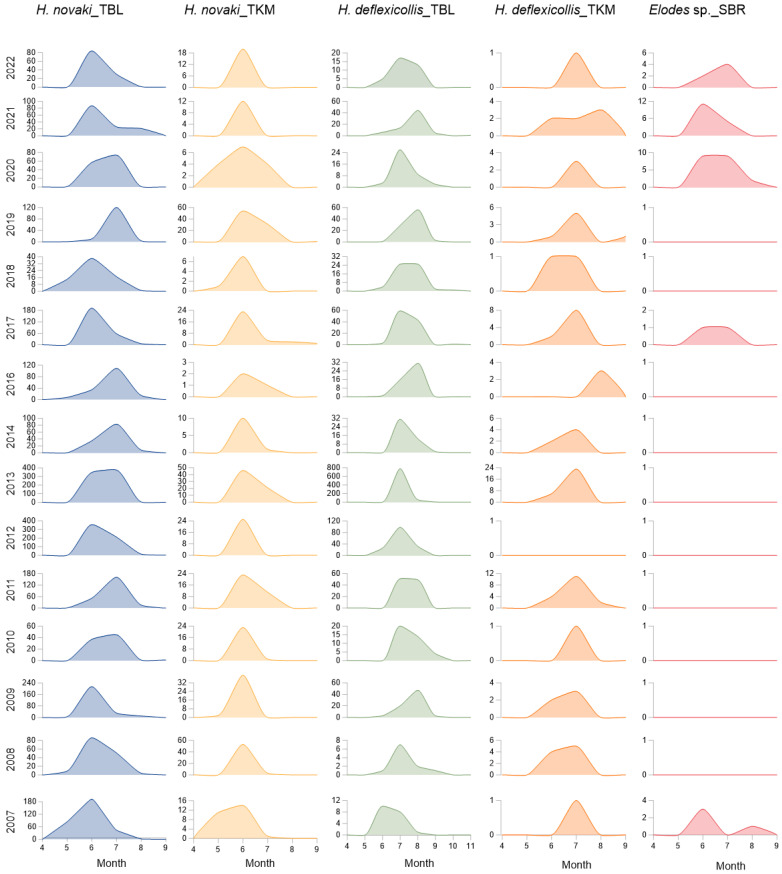
Seasonal dynamic in scirtids’ emergence in Plitvice Lakes NP during 15 years at three study sites. Abbreviations of the sites as in Table 1. *Y*-axis: the number of individuals. Only the months in which scirtids were recorded are shown.

The highest abundance of emerged scirtids was recorded In summer. At the spring of the Bijela rijeka river, the earliest time of emergence for the *Elodes* sp. was recorded in June in 2007, 2017, 2020 and 2021. The latest time of emergence was found to be in September in 2007 and 2020. In 2017 and 2021, their emergence had ended already in July. At the tufa barriers, the earliest time of emergence for *Hydrocyphon novaki* was recorded in May, and the highest abundance was reached in June. At the Kozjak–Milanovac tufa barrier, the emergence of the species ended before July in 2009, 2012, 2018 and 2021. The latest time of emergence was in August, except in 2010 and 2012, when the species was also recorded in September. The emergence of *H. deflexicollis* mostly started in June, except in 2010, 2011 and 2014, when the species was recorded in July. The month when the species reached the highest abundance varied during the study period—June, July or August. The emergence of the species mostly ended in September, except in 2007, 2011, 2012 and 2016, when it was over in August. Exceptionally, in 2017 and 2018, a specimen was also recorded in October and even in November in 2021 (Figure 4).

### 3.3. Environmental Characteristics and Scirtids

The environmental characteristics of the study area are presented in Table 1. The parameters measured in the spring area showed less variation compared to those at the tufa barriers. Thus, both the highest and lowest water temperatures were measured at the Kozjak–Milanovac barrier (22.95 °C and 1.66 °C, respectively). The water alkalinity and conductivity decreased downstream, while pH and oxygen saturation were highest at the downstream Kozjak–Milanovac site. The nutrient concentrations were mostly low at all the study sites (Table 1).

**Figure 5:**
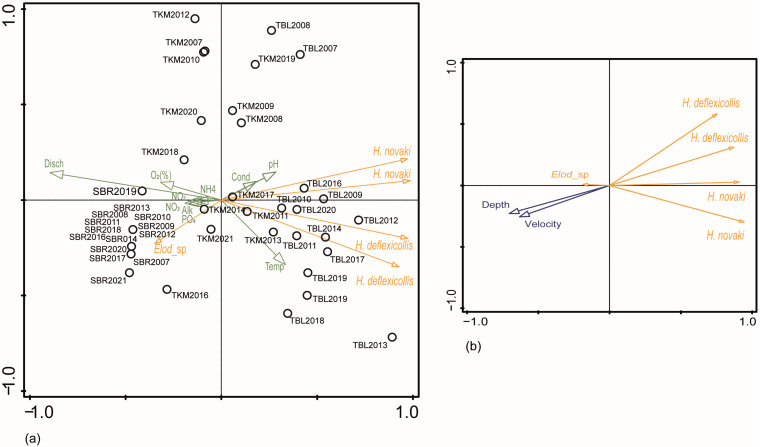
Principal component analysis of scirtids in Plitvice Lakes NP: (**a**) at three study sites; (**b**) at microhabitats. Abbreviations: Temp: temperature, Disch: water discharge, Cond: conductivity, Alk: alkalinity; abbreviations of other parameters and sites as in Table 1.

When analysed at the microhabitat level, Spearman’s coefficient showed that the abundance of the two *Hydrocyphon* species correlated with the current velocity (*H. novaki*: ρ = 0.228, ρ = 0.197, *p* < 0.001; *H. deflexicollis*: ρ = 0.152, *p* < 0.05). At the Labudovac tufa barrier, for example, the highest abundance of beetles was recorded in 2013 (especially in microhabitat P7) when the current velocity was low. In contrast, scirtids were less abundant in the gravel and sand substrate when the water velocity was higher, as was the case at both barriers in 2014. The *Elodes* sp. showed a preference for lower water velocities (ρ = −0.123, *p* < 0.05). The principal component analysis at the microhabitat level, which included three taxa and two parameters (water depth and current velocity), confirmed these results with eigenvalues for the first two axes of 0.7764 and 0.0992 and a cumulative explanation for the variation of 87.56% (Figure 5).

**Figure 6:**
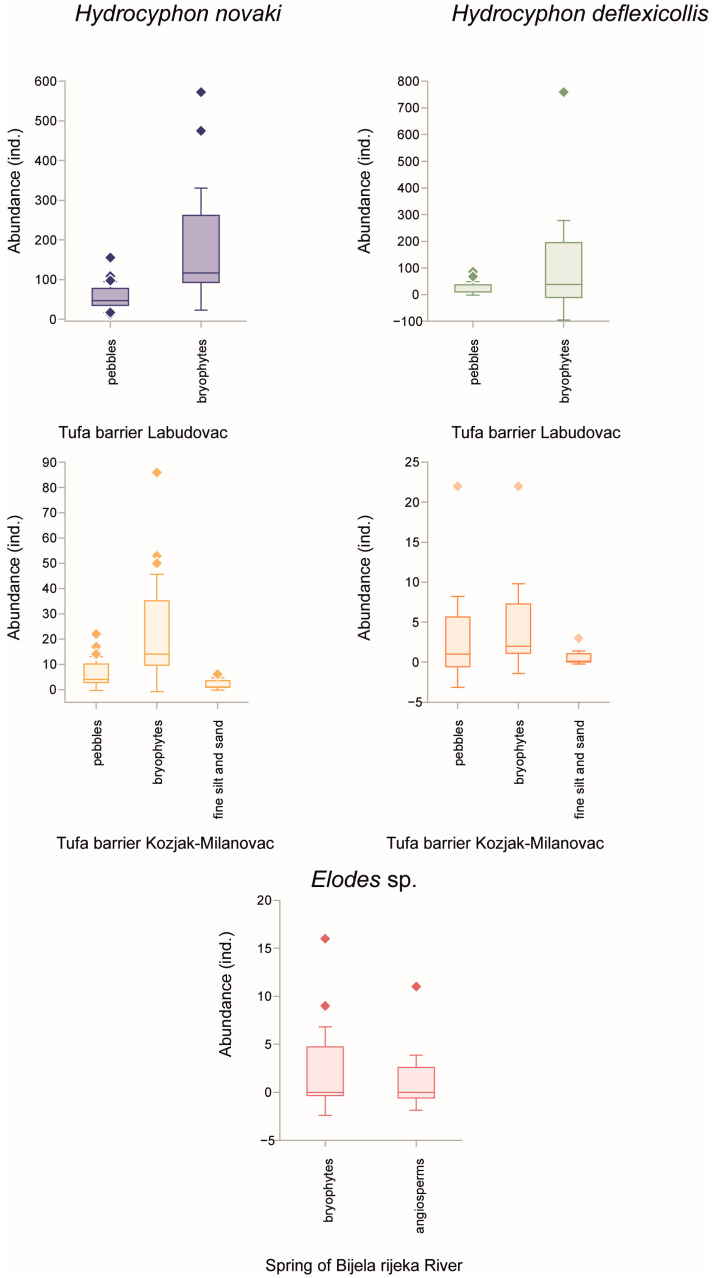
Box-whisker plots of the abundance of family Scirtidae during 15 years in Plitvice Lakes NP on different substrate types. The box edges show the confidence level (95%), the centre line the median value and the whiskers the standard deviation.

At the Labudovac tufa barrier, the bryophyte substrate enabled a higher abundance of scirtids compared to the gravel and sand substrates. At the Kozjak–Milanovac tufa barrier, where three main substrate types were defined, byropyhtes again proved to be the prefered substrate type for both *Hydrocyphon* species. Angiosperms, on the other hand, encountered the lowest abundance of scirtids. At the spring of the Bijela river, the *Elodes* sp. was most frequently found in the bryophytes, while it was absent in gravel and sand (Figure 6).

Based on Spearman’s correlation coefficient for scirtids and the site-level environmental characteristics, both sexes of both *Hydrocyphon* species were positively correlated with water temperature (*H. novaki*: male ρ = 0.385, female ρ = 0.325; *H. deflexicollis*: ρ = 0.358, ρ = 0.393, *p* < 0.01). Both sexes of *H. novaki* and males of *H. deflexicollis* were also positively correlated with pH (*H. novaki* male ρ = 0.326, female ρ = 0.389; *H. deflexicollis* male ρ = 0.319, ρ = −0.783, *p* < 0.001). On the contrary, a strong negative correlation was recorded for both species with water discharge (*H. novaki* male ρ = −0.886, female ρ = −0.891; *H. deflexicollis* male ρ = −0.842, ρ = −0.783, *p* < 0.001) and with nutrients—i.e., nitrates (*H. novaki* male ρ = −0.512, female ρ = −0.613; *H. deflexicollis* male ρ = −0.519, ρ = −0.522, *p* < 0.001). Additionally, the genus *Elodes* showed no significant correlation with the environmental parameters.

Three taxa and ten physico-chemical parameters in 19 microhabitats were included in the principal component analysis. On the F1 × F2 ordination plot (Figure 5), the eigenvalues for the first two axes were 0.9171 and 0.0481 with a cumulative explanation for the variation of 96.52%. Both axes showed the strongest correlation with water temperature (R = −0.3345; R = −0.3389). Based on ordination, the *Elodes* sp. showed the strongest positive correlation with alkalinity, orthophosphates and oxygen saturation and a negative one with water temperature. Water temperature was the most important factor for both sexes of *H. deflexicollis*. Females and males of *H. novaki* showed a positive correlation with conductivity and pH.

## 4. Discussion

### 4.1. Faunal Asemblage and Distribution

The family Scirtidae has been studied from the perspective of taxonomy [55,56] and specific ecological traits [57,58,59], but its emergence patterns and environmental drivers have been poorly studied. Therefore, the present study is the first ever comprehensive long-term study of the biological and ecological data and diversity patterns, as well as the emergence aspects, of this family.

Matoničkin et al. [60] and Mičetić Stanković et al. [11,61] reported the occurrence of the species *Hydrocyphon deflexicollis* and genus *Elodes* in Plitvice Lakes NP, which is consistent with our study.

In the present study, another species for this area, *Hydrocyphon novaki*, was also reported. The longitudinal zonation of the family is confirmed [11], as the genus *Elodes* inhabits only the spring area, while *Hydrocyphon* lives in the downstream sections. A high abundance of scirtids at the tufa barriers is also confirmed, with the highest numbers not at the Kozjak–Milanovac tufa barrier but at the Labudovac tufa barrier. These high abundances can be explained by the presence of various microhabitats typical of tufa barriers that constitute “freshwater coral reefs” [62,63,64]. Moreover, organically enriched water pours over these barriers, allowing the development of a rich phytobenthic layer (see more in [65]) and the accumulation of detritus, which is the preferred food for *Hydrocyphon* species [66,67]. Bryophytes proved to be the preferred substrate type for both genera of scirtids, which is consistent with previous studies [2,11,68].

### 4.2. Sex Ratio and Emergence Patterns

The sex ratio of water beetles is generally poorly known and studied [69]. The low number of *Elodes* representatives could have been caused by an inadequate sampling method. In particular, Klausnitzer [66] noted that their pupation takes place above the water surface. It is noteworthy that the authors carried out additional sampling of male adults at the spring of the Bijela rijeka river, but without success. The sex ratio of *Hydrocyphon* varied greatly during the study period, with males predominating, which was also suggested by Cuppen [57]. Juliano [70] studied dytiscids and reported that males emerge first to secure copulation with females, who emerge later. The same pattern was observed in our study.

Our results are consistent with the idea that representatives of *Hydrocyphon* are univoltine [66]. Emergence occurs in the summer months [57], with water temperature being the limiting factor [71].

### 4.3. Environmental Characteristics and Scirtids

The abiotic parameters showed a longitudinal change in their values, as has been already confirmed in various studies (e.g., [11,32,33,40,72]). Furthermore, the water is an oligotrophic hydrosystem rich in carbonates [24]. Water temperature proved to be the most important environmental factor for the emergence of the family Scirtidae in the studied area. This is already known for many other aquatic insects groups inhabiting Plitvice Lakes NP [32,33,35,36,40,72,73,74]. The water temperature influences the individual growth rate, metabolism and thus the timing of emergence of aquatic insects [75,76,77]. In 2013, a considerably higher abundance was reported at the Labudovac tufa barrier, possibly due to the warm winter of 2012/2013, when the minimum water temperatures were quite high [72]. Since *Hydrocyphon* overwinters in the larval stage [66], higher temperatures affected their growth and reproductive success, as well as their timing and emergence patterns, in the coming year. Furthermore, this high increase in their abundance can be explained by the recovery of the population after the great drought of 2011/2012 [39]. The heavy loss of precipitation with significantly reduced water discharge was accompanied by the absence of differences in water temperature, as this ecosystem is predominantly fed by groundwater. Moreover, water discharge proved to be one of the most important factors in determining the abundance of scirtids, as already confirmed in previous studies for other aquatic insects in the same habitats [38]. The latter study showed that the duration of precipitation and snow cover changes in the area led to strong seasonal fluctuations in water discharge, which of course strongly affects benthic organisms. The current velocity apparently plays an important role in the assemblage and composition of scirtids, which was already suggested by Mičetić Stanković et al. [11] in the same area. These results are in accordance with studies on scirtids in other freshwater habitats [31,57,78]. Moreover, scirtids were less abundant during the years when the current velocities were higher in the microhabitats with gravel and sand than during the years when the velocity was lower.

In conclusion, our study represents the first comprehensive study of the emergence patterns of the still-overlooked water beetles of the family Scirtidae. Many of its results are the first to be reported for scirtids and thus contribute to our overall knowledge on water beetle biodiversity in this part of Europe. The family proved its potential to be used as an indicator of stream zonation. The sex ratio is in favour of males in the spring and early summer, while it shifts in favour of females towards the end of their emergence. Their emergence shows a seasonal character, as the highest intensity is recorded in summer. Primarily water temperature, secondarily current velocity and oxygen saturation played an important role in the abundance and distribution of scirtids. This study is not only a basis for further studies on the emergence of water beetles but also for monitoring today’s vulnerable karst freshwater ecosystems in the light of ongoing climate change.

## Figures and Tables

**Table 1:** Characteristic of study sites in Plitvice Lakes NP. Abbreviations: SBR: spring of the Bijela rijeka river, TBL: tufa barrier Labudovac, TKM: tufa barrier Kozjak–Milanovac.

Site	SBR	TBL	TKM
Latitude	N 44°50′05″	N 44°52′17″	N 44°53′39″
Longitude	E 15°33′43″	E 15°35′59″	E 15°36′32″
Altitude (m)	720	630	546
Water temperature (°C)			
min	7.2	1.663	1.67
max	8.4	20.63	22.95
O_2_ (%)			
min	65.21	59.7	72.04
max	106.3	139.2	130.5
pH			
min	6.8	7.95	7.98
max	8.9	8.63	8.52
Alkalinity (mg CaCO_3_ L^−1^)			
min	242.2	180.94	159.5
max	302.3	270.5	226.5
Conductivity (µS cm^−1^)			
min	384	348	323
max	528	445	415
NO_3_ (mg N L^−1^)			
min	0	0.01	0.01
max	1.58	1.06	1.14
NO_2_ (mg N L^−1^)			
min	0	0	0
max	0.002	0.008	0.01
NH_4_^+^ (mg L^−1^)			
min	0	0	0
max	0.2	0.22	0.103
PO_4_^−^ (mg L^−1^)			
min	0	0	0
max	0.049	0.07	0.255
Water discharge (m^3^ s^−1^)			
min	0.15	1.74	1.4
max	1.07	5.58	4.22
Emergence trap/substrate type			
P1	gravel and sand	pebbles	pebbles
P2	gravel and sand	pebbles	fine silt and sand
P3	bryophytes	bryophytes	pebbles
P4	angiosperms	pebbles	bryophytes
P5	bryophytes	bryophytes	bryophytes
P6	angiosperms	bryophytes	fine silt and sand
P7	-	bryophytes	-
Current velocity (m^3^ s^−1^)			
min	0	1.2	0
max	37	50.56	48.66
Depth (m)			
min	0.06	0.08	0.08
max	0.25	0.45	0.48

## Data Availability

The data supporting the reported results can be provided upon contacting the corresponding author.
